# Strengths, weaknesses, opportunities, and threats analysis of integrating the World Health Organization patient safety curriculum into undergraduate medical education in Pakistan: a qualitative case study

**DOI:** 10.3352/jeehp.2017.14.35

**Published:** 2017-12-28

**Authors:** Samreen Misbah, Usman Mahboob

**Affiliations:** 1 Department of Community Medicine, Army Medical College, National University of Medical Sciences, Rawalpindi, Pakistan; 2 Institute of Health Professions Education and Research, Khyber Medical University, Peshawar, Pakistan; Hallym University, Korea

**Keywords:** Curriculum, Medical education, Pakistan, Patient safety

## Abstract

**Purpose:**

The purpose of this study was to conduct a strengths, weaknesses, opportunities, and threats (SWOT) analysis of integrating the World Health Organization (WHO) patient safety curriculum into undergraduate medical education in Pakistan.

**Methods:**

A qualitative interpretive case study was conducted at Riphah International University, Islamabad, from October 2016 to June 2017. The study included 9 faculty members and 1 expert on patient safety. The interviews were audiotaped, and a thematic analysis of the transcripts was performed using NVivo software.

**Results:**

Four themes were derived based on the need analysis model. The sub-themes derived from the collected data were arranged under the themes of strengths, weaknesses, opportunities, and threats, in accordance with the principles of SWOT analysis. The strengths identified were the need for a formal patient safety curriculum and its early integration into the undergraduate program. The weaknesses were faculty awareness and participation in development programs. The opportunities were an ongoing effort to develop an appropriate curriculum, to improve the current culture of healthcare, and to use the WHO curricular resource guide. The threats were attitudes towards patient safety in Pakistani culture, resistance to implementation from different levels, and the role of regulatory authorities.

**Conclusion:**

The theme of patient safety needs to be incorporated early into the formal medical education curriculum, with the main goals of striving to do no harm and seeing mistakes as opportunities to learn. Faculty development activities need to be organized, and faculty members should to be encouraged to participate in them. The lack of a patient safety culture was identified as the primary reason for resistance to this initiative at many levels. The WHO curriculum, amended according to local institutional culture, can be implemented appropriately with support from the corresponding regulatory bodies.

## Introduction

Despite remarkable improvements in health care outcomes due to scientific innovations in modern medicine, studies from numerous countries have shown that hospitalized patients are at risk of adverse outcomes [1]. Medical errors in health care were not considered to be a preventable cause of morbidity and mortality until a foundational report on the topic was issued in 1999, followed by subsequent reports that highlighted the need for a safer system of care and the provision of quality health care as a universal concern [2]. Harm to patients is foreseeable, and to acknowledge it, health care workers and organizations need to learn from past errors to bypass imminent errors. To compete as part of an increasingly complex health care system, our future health care leaders require the competencies of patient safety knowledge, fundamental skills, and behaviors necessary to reduce patient harm. Health care professionals receive comparatively little education regarding harm reduction, and the limited patient safety knowledge among medical trainees that has been revealed by several studies means that effective educational interventions are needed to target this deficiency. Short patient safety pilot programs have shown that the continued implementation of a patient safety curriculum prepares medical students to better practice [3]. The World Health Organization (WHO) developed patient safety as a specialized discipline, and in 2009 a comprehensive guide was introduced for worldwide patient safety education implementation in medical schools [1,4]. This comprehensive guide focuses on 10 topics derived from patient safety education in Australia, and an additional topic selected by the WHO was to support learning in infection control. Still, few medical schools have implemented undergraduate patient safety curricula, as many factors have hampered patient safety education [5]. A study conducted in Pakistan found important reasons for violence against health care professionals to be unreasonable expectations, unexpected outcomes, communication failure, and human errors, in a context where medical graduates reliably lack the necessary knowledge, attitudes, and practical skills to provide safe care [6]. Being unfamiliar with the new literature on systems thinking and quality improvement methods, medical educators did not recognize that patient safety skills can be taught and were uncertain about how to integrate patient safety into the existing curriculum [5]. In Pakistan, patient safety is a relatively new concept and medical universities are therefore unsure of how to incorporate patient safety into their existing curricula. Thus, we planned a needs analysis study using the strengths, weaknesses, opportunities, and threats (SWOT) approach to ask faculty about their views on integrating the WHO patient safety curriculum into undergraduate medical education in Pakistan.

## Methods

A qualitative interpretive case study was conducted at Riphah International University in Islamabad, Pakistan from October 2016 to June 2017. It was described according to the Consolidated Criteria for Reporting Qualitative Research (COREQ) 32-item checklist for qualitative studies [7].

### Research team and reflexivity

All interviews were conducted by the corresponding author, Samreen Misbah masters of health professions education [MHPE] trainee, (masters of public health [MPH] and a bachelor of medicine and bachelor of surgery [MBBS]). Samreen Misbah MHPE trainee at the time of study, has MPH in community medicine, and MBBS in medicine. Usman Mahboob is a Doctor of philosophy in Health Professions Education with an MPH in community medicine and an MBBS in medicine. Both authors were assistant professors at the time of the study. Samreen Misbah is female and Usman Mahboob is male. They were both trained in health professions education. Their credibility was ensured by spending the maximum possible time at the inquiry site, and the researchers allocated time for the participants to identify the salient features of the context and problem, and explained the purpose of the interviews. One to two meet ings were conducted prior to the interviews, and specific interview times were booked. Participants were given details about the researcher and the reasons why they were selected for an interview about their perceptions regarding the need analysis of integration of the WHO patient safety curriculum. Detailed information about the research project, which was conducted for the partial fulfillment of MHPE training, was also provided to them.

### Study design

The case study methodology and grounded theory analysis under interpretative assumptions were combined to construct a suitable research methodology [8]. A total of 10 participants were included in the study. Information-rich cases through purposeful sampling were selected from a wide variety of sources. An introduction to the study, along with the curriculum, was sent via email to all participants explaining the purpose of the research project that was conducted for partial fulfillment of MHPE training, with the goal of conducting a need analysis of integration of the WHO patient curriculum. The first author booked time for face-to-face interviews. Nine senior faculty members from the basic and clinical sciences with at least 5 years of teaching experience, experience with teaching an integrated curriculum, and a background in health professional education were selected. These participants were working as full, associate, or assistant professors in their respective departments. A professor of medicine working as the principal and director of the Riphah Institute of Healthcare Improvement and Safety was also included, as this had been recently launched at the university. Faculty who did not fulfill the criteria or were not willing to participate were excluded (Table 1). All participants who were approached co-operated with the interviews, and none dropped out. Interviews were arranged in participants’ workplaces. Only the participant was present, and each interview was conducted in a single session. The theoretical sampling was based on analyses of collected data from previous interviews that guided the researcher regarding future data collection, and the sample size was not fixed. The details of the participants and the date and time of the interviews are shown in Table 1.

The Pakistan Medical and Dental Council (PMDC) curriculum for the MBBS degree currently followed in medical schools was analyzed to identify learning outcomes related to patient safety topics. Questions for the interviews were based on a literature review and were sent to 5 experts in medical education with at least 3–5 years of experience, and to a Korean researcher with expertise in the patient safety curriculum. A pilot interview was done with a faculty member at the same university, and finally, 8 open-ended questions were selected. The interview guide with the final questions was e-mailed to the participants prior to the interviews. The interviews were completed in a single session and were not repeated. Two sources were used to audiotape the interviews; few measures were taken to avoid background sounds and interruptions, and field notes were taken during the sessions. The time for each interview was estimated to be approximately 50 minutes.

The process of data collection was stopped when the theoretical base was saturated [9]. All raw data, audiotaped interviews, and interview transcripts were sent to the participants to confirm their accuracy and to ensure credibility.

### Analysis and findings

**Fig. 1.**
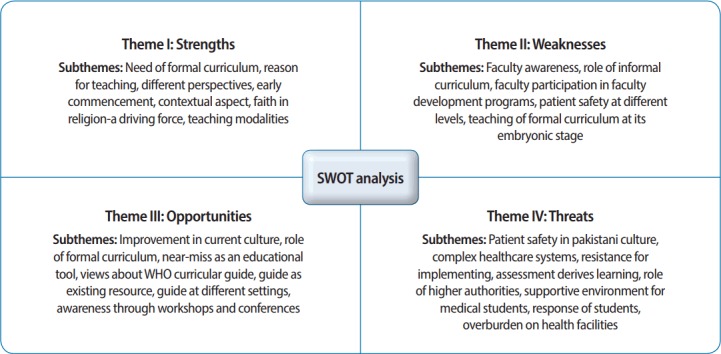
Themes and sub-themes identified for integration of the World Health Organization patient safety curriculum. SWOT, strengths, weaknesses, opportunities, and threats.

The transcripts were also scrutinized by the second author, who had expertise in medical education, and the data were coded by both authors, who reviewed and discussed the results. As a key process of grounded theory, coding began early after the first interview and open coding was done by categorizing data. Axial coding was done to relate the 4 categories of strengths, weaknesses, opportunities, and threats to the subcategories. Finally, by selective coding, core categories were identified for the SWOT analysis of integration of the WHO patient safety curriculum. Individual transcripts were read line by line; salient features related to research objectives and research questions were identified, chunks of the text were labeled with codes, and hierarchies of nodes were organized. Long lists of codes from the data were collected and merged to make a total of 27 codes. Categories were created by combining several codes. Four themes for the SWOT analysis of integration of the WHO patient safety curriculum into undergraduate medical education in Pakistan were derived based on the need analysis model. The sub-themes derived from the collected data were arranged under the themes (Fig. 1). Data analysis was done in NVivo software ver. 11.0 (QSR International, Doncaster, Vic, Australia), for which the corresponding (first) author received training. The transcribed interviews were imported into the software and manual data analysis was also done as needed. Attribute codes were given to the participants to maintain anonymity. Additional information suggested by the participants after they checked the transcript was incorporated in the final report.

### Quality assurance

This research was described according to the COREQ guidelines. Data triangulation from 2 sources of evidence (audiotaped interviews and field notes) was performed. Raw material was saved for future reference and as an audit trail. All participants were asked to review the interview transcripts and any additional information, if suggested, was incorporated into the final report. A case study database was created and stored in a manner that can be retrieved in several raw and process stages. Participants from different clinical and basic sciences were selected through purposeful sampling to maximize the collected information and to enhance transferability to other similar contexts [8].

### Ethical approval

Ethical clearance for the study was granted by the Institutional Review Committee of Riphah International University (IRB no. Riphah/ERC/17/0211) after receiving informed consent from the participants.

## Results

A total of 10 participants were interviewed, which took more than 100 hours of transcription time, and the total word count of all interviews was 40,096. Individual transcripts were read line by line; salient features related to research objectives and research questions were identified, and chunks of the text were labeled by making codes and saved in the storage area of nodes. Quotations from the participants to illustrate the themes were identified by attribute codes and are presented in Tables 2–5. The collected data show consistent findings and interpretations of both major and minor themes. The major theme of “strengths” showed the positive internal attributes of the system identified by the participants that were under the participants’ control and in a suitable condition for implementing the curriculum (Table 2). The theme of “weaknesses” represented internal areas of the system that lacked competencies as acknowledged by the participants, could destabilize implementation of the curriculum, were considered completely under control, and could be improved (Table 3). The third major theme, “opportunities,” described positive external factors that were beyond our control but could open up possibilities for implementing the curriculum if taken advantage of (Table 4). Finally, the main theme of “threats” depicted threats identified by the participants as external factors that were also beyond our control and for which a plausible plan to combat the accompanying risk was possible (Table 5).

Sub-themes under each major theme are described in detail in Fig. 1. The strengths identified included the need for a formal patient safety curriculum and its early integration into the undergraduate program. The weaknesses were faculty awareness and participation in development programs. The opportunities were an ongoing effort to develop an appropriate curriculum, to improve the current culture of health care, and to use the WHO curricular resource guide. The threats were attitudes towards patient safety in Pakistani culture, resistance to implementation from different levels, and the role of regulatory authorities.

## Discussion

The purpose of this qualitative research was to highlight patient safety as a neglected component of the appropriate education and training of health care professionals that has recently become an area of concern. However, curriculum developers are uncertain about the teaching and integration of the topic. In the present study, we conducted a needs analysis to understand the insights of faculty members at a university in Pakistan. Different aspects of the internal and external factors for integration of the WHO patient safety curriculum into medical education were emphasized using the SWOT approach.

The current study suggested that patient safety education needs to be disseminated as a formal curriculum that may be introduced gradually and progressively. Presently, individual topics are communicated to students in a piecemeal fashion, with the consequence that the recipients are not sensitized to the importance of the topic. A brief lecture-based program may be sufficient to bring short-term positive changes, but for a smooth introduction of patient safety teaching into the undergraduate curriculum, a formal explicit curriculum must be used [10].

The WHO curricular guide was considered quite flexible and easy to implement by the study participants. They recommended it as a guideline that can be used according to national health care facilities and the culture on the ground. An evaluation study of the WHO patient safety curricular guide that was conducted to investigate the categorical implementation of explicit patient safety education reported that the curriculum guide was a helpful resource. However, the successful implementation of a curriculum requires the faculty to be familiar with the material, for the necessary time to be invested into developing the curriculum in the local context, for sufficient leadership and commitment to be present, and for the trainers to be trained [11].

The main weaknesses identified by participants were the awareness of the patient safety curriculum among faculty members, who must be trained first, and faculty development activities to raise their competencies in this area. Teaching the topics in a modular fashion was considered promising for implementation because integration of the entire curricular guide may result in several problems and place resource constraints on an institution. Participants were in favor of focusing on the effective implementation of the curriculum at a single institution as a pilot study that can be implemented at other institutions after seeing the results and requirements. These considerations suggest that the curricular guide should be tailored according to the culture of each institution, and that a wide range of teaching methods should be used to teach patient safety topics, ranging from whole-group lectures to small-group discussions and simulation- or clinical-based activities, as described in other studies [12]. All the teaching strategies that are used in present-day teaching need to be incorporated to support this part of the curriculum as well. Even exposure to field work and community problems can be an excellent source of lessons about safety hazards and the management thereof.

The faculty members surveyed in the present study believed that awareness sessions need to be conducted for students to understand the benefits of practicing patient safety culture for their own safety and health. Students are considered to be an excellent source for recognizing errors, and they can be trained accordingly. Despite their limited baseline knowledge of this topic, evaluation studies have found students to have a positive attitude towards the curriculum and that they have reported increases in their knowledge after teaching sessions [13]. Most medical errors cannot be attributed to individual negligence. The current study revealed patient safety issues at many levels of health care personnel, including doctors, nurses, paramedical staff, as well as at shift changes of nurses and due to lack of counseling by doctors. Miscommunication between health care personnel or with patients may be responsible for errors. A safer environment for patients also produces a safer environment for health care providers. The participants of the study stated the opinion that the current culture of health care is quite resistant to patient safety culture because everyone is not used to it, but that once patient safety culture is implemented, the resistance will decrease [14]. In response to the high rate of adverse events, the Patient Safety Friendly Hospital Initiative was launched in 7 developing countries of the Eastern Mediterranean region, and highlighted the convincing suggestion that the implementation of patient safety standards in these hospitals increased the level of awareness of participating hospitals and patients [15].

The main reason in this study for teaching patient safety education was encouraging students to avoid errors and to consider mistakes to be opportunities to learn how to provide quality medical care. Although physicians have been considered reluctant partners in reporting errors, they have shown willingness to share near misses with their institutions to avoid future errors. Participants suggested that there must be an assessment of this part of the curriculum that differentiates between learners and non-learners. However, in routine clinical settings in hospital wards, facilitators may observe and give feedback to the students in an informal way, thereby inculcating these principles as a part of ethical behavior. Assessment facilitates learning; however, a distinction can be made between assessments for learning and assessments of learning.

Some of the conclusions of the study were limited by time constraints. Although the PMDC is the main governing body for decisions about the medical education curriculum, representatives from it were not approached. Further research into implementing the curriculum in this context may help to complete the picture. The participants in this study considered patient safety to be the most neglected area of education and training, and a complex topic, for which a formal curriculum starting early in the medical education process is required. The main reason for teaching patient safety was the goal to do no harm. The faculty members were not fully aware of the need to train and participate in faculty development programs; patient safety issues occur at many levels and resistance to this curriculum was identified as being due to the lack of a patient safety culture. Once the true intentions of implementation are known, resistance will decrease. In quality medical care, a mistake is an opportunity to learn. The necessary amendments in the curriculum should be made according to our culture on the ground and level of literacy regarding these issues. This study emphasized that as patients are becoming aware of their rights, students must learn about patients’ rights and the necessity thereof. The WHO patient safety curriculum can be implemented if the PMDC makes it mandatory for medical schools and hospitals to receive assistance in developing patient safety curricula.

## Figures and Tables

**Table 1.** Respondents’ demographics

No.	Respondents’ID	Date of interview	Gender	Department	Credentials
1	FW	20-3-2017	Female	Gynecology and obstetrics	MBBS, FCPS, JHPE
2	KFD	20-3-2017	Male	Surgery	MBBS, FCPS, PGT, MHPE
3	FQ	21-3-2017	Male	Medicine	MBBS, FCPS, PGT, MHPE (trainee)
4	MA	27-3-2017	Female	Pathology	MBBS, FCPS, MHPE
5	AB	28-3-2017	Male	Community medicine	MBBS, MPH, PGT, MHPE
6	UZ	30-3-2017	Female	Pharmacology	MBBS, M. Phil
7	SH	30-3-2017	Female	Physiology	MBBS, M. Phil, PhD
8	MU	7-4-2017	Male	Surgery	MBBS, FCPS, MCPS (medical education)
9	SM	10-4-2017	Female	Pediatrics	MBBS, FCPS
10	MUR	13-4-2017	Male	Institute of healthcare improvement and safety	MBBS, DTCD, MCPS, DIMS, CPQP, CPHQ

MBBS, bachelor of medicine and bachelor of surgery; FCPS, fellow of the College of Physicians and Surgeons; JHPE, joint Human performance enhancement; PGT, post graduate trainee; MHPE, masters of health professions education; MPH, master of public health; M. Phil, master of philosophy; PhD, doctor of philosophy; MCPS, member of College of Physicians and Surgeons (Canada); DTCD, diploma in tuberculosis and chest diseases; DIMS, Doctors’ Institute of Medical Sciences; CPQP, Certified Pharma Quality Professional; CPHQ, Certified Professional in Healthcare Quality.

**Table 2.** Summary of the major theme of strengths and the sub-themes that emerged from the analysis of faculty interviews, along with representative quotations from interview transcripts

Sub-themes	Description	Frequency	References	Representative quotes
Need for a formal curriculum	Following a formal curriculum is considered more effective	9	31	AB: “I think it is the right time now, if you want to keep pace in between the developing and developed world”.
Reason for teaching	Doing no harm; safety of patients for ourselves and for our coming generation	10	26	FW: “It’s just like if a jet falls we are also worried, and 200, 300 people are dying, so it’s like 7 jets falling in a week with so many patients dying because of lack of patient safety”.
Early integration of patient safety education	Fundamental information should establish background knowledge; thereafter, specific knowledge should be imparted	8	18	SM: “Yes I will say that it can start from the very first year because this is the work for which they have come.”

**Table 3.** Summary of the major theme of weaknesses and sub-themes that emerged from the analysis of faculty interviews, along with representative quotations from interview transcripts

Sub-themes	Description	Frequency	References	Representative quotes
Faculty awareness	Faculty and system must be aware of its applicability in our social context	10	42	UZ: “Weaknesses are the training of the trainers first; we must have the trained person who will train the trainers”.
Role of the informal curriculum	Topic not taught as an explicit component of the curriculum	9	27	UZ: “In informal way I taught them how you can prevent spread of infections, because I know I don’t have any formal curriculum.”
Participation in faculty development programs	Many faculty development activities are required to train faculty to train their students	10	19	MA: “We need to have faculty development before that, so we are all on the same page, that is the weakness and challenge we have”.

**Table 4.** Summary of the major theme of opportunities and sub-themes that emerged from the analysis of faculty interviews, along with representative quotations from interview transcripts

Sub-themes	Description	Frequency	References	Representative quotes
Role of the formal curriculum	Until the curriculum is conducted in a structured way and implemented officially, it cannot be effective.	9	29	KFD: “The biggest opportunity that exists for us is an ongoing effort for a proper curriculum designing at the national level. Anything positive would be rightly implemented in our evolving curriculum.”
Learning from errors, near misses as an educational tool	In quality medical care, a mistake is an opportunity to learn.	10	27	KFD: “We have to remove the threat component from the recognition of errors; that can be to the professional integrity, personal identity or deficiency of the existing system of practice.
Views about WHO curricular guide	Opinions regarding the WHO patient safety curriculum	10	19	MA: “No, everybody needs to tailor it according to their needs, their facilities, and whatever is happening over there. One curriculum does not fit all.”

WHO, World Health Organization.

**Table 5.** Summary of the major theme of threats and sub-themes that emerged from the analysis of faculty interviews, along with representative quotations from interview transcripts

Sub-themes	Description	Frequency	References	Representative quotes
Patient safety in Pakistani culture	To create a patient safety culture in our environment, we have to start from somewhere	10	30	MU: “It is not all or none process. We do not expect that today we implement and tomorrow these guidelines are followed.
Resistance to implementation from different levels	The current culture is quite resistant because everyone is not used to it	10	22	SH: “When there is check and balance there is threat to a number of people who are not doing things right.”
Role of regulatory authorities in implementation	If the Pakistan Medical and Dental Council implements it and makes it compulsory, then the whole medical establishment will be involved	7	18	KFD: “Some initiative needs to be taken at the level of our national regulatory authorities which are the policy makers of health care provision in Pakistan.”

## Supplementary material

Supplement 1. Audio recording of the abstract.

## References

[1] Walton M, Woodward H, Van Staalduinen S, Lemer C, Greaves F, Noble D, Ellis B, Donaldson L, Barraclough B (2010). Expert Group convened by the World Alliance of Patient Safety, as Expert Lead for the Sub-Programme. The WHO patient safety curriculum guide for medical schools. Qual Saf Health Care.

[2] Myung SJ, Shin JS, Kim JH, Roh H, Kim Y, Kim J, Lee SI, Lee JH, Kim SW (2012). The patient safety curriculum for undergraduate medical students as a first step toward improving patient safety. J Surg Educ.

[3] Wong BM, Etchells EE, Kuper A, Levinson W, Shojania KG (2010). Teaching quality improvement and patient safety to trainees: a systematic review. Acad Med.

[4] World Alliance for Patient Safety (2009). WHO patient safety curriculum guide for medical schools [Internet]. http://www.who.int/patientsafety/information_centre/documents/who_ps_curriculum_summary.pdf.

[5] Bohomol E, Cunha IC (2015). Teaching patient safety in the medical undergraduate program at the Universidade Federal de Sao Paulo. Einstein (Sao Paulo).

[6] Jawaid SA (2016). Prevention and intervention strategies to check increasing violence against Healthcare Facilities and Healthcare Professionals. Pak J Med Sci.

[7] Tong A, Sainsbury P, Craig J (2007). Consolidated criteria for reporting qualitative research (COREQ): a 32-item checklist for interviews and focus groups. Int J Qual Health Care.

[8] Halaweh M, Fidler C, McRobb S (2008). Integrating the grounded theory method and case study research methodology within is research: a possible “road map” [Internet]. http://aisel.aisnet.org/icis2008.

[9] Yazan B (2015). Three approaches to case study methods in education: Yin, Merriam, and Stake. Qual Rep [Internet]. http://www.nova.edu/ssss/QR/QR20/2/yazan1.pdf.

[10] Leung GK, Patil NG (2010). Patient safety in the undergraduate curriculum: medical students’ perception. Hong Kong Med J.

[11] Patey R, Flin R, Ross S, Parker S, Cleland J, Jackson J, Moffat M, Thomson A (2011). WHO patient safety curriculum guide for medical schools: evaluation study: report to WHO Patient Safety Programme August 2011 [Internet]. http://www.who.int/patientsafety/education/curriculum/PSP_Eval_Study_Report-2011_March-2012.pdf.

[12] Leung GK, Patil NG, Ip MS (2010). Introducing patient safety to undergraduate medical students: a pilot program delivered by health care administrators. Med Teach.

[13] Nie Y, Li L, Duan Y, Chen P, Barraclough BH, Zhang M, Li J (2011). Patient safety education for undergraduate medical students: a systematic review. BMC Med Educ.

[14] Weaver SJ, Lubomksi LH, Wilson RF, Pfoh ER, Martinez KA, Dy SM (2013). Promoting a culture of safety as a patient safety strategy: a systematic review. Ann Intern Med.

[15] Siddiqi S, Elasady R, Khorshid I, Fortune T, Leotsakos A, Letaief M, Qsoos S, Aman R, Mandhari A, Sahel A, El-Tehewy M, Abdellatif A (2012). Patient Safety Friendly Hospital Initiative: from evidence to action in seven developing country hospitals. Int J Qual Health Care.

